# Effect of different dietary omega-3/omega-6 fatty acid ratios on reproduction in male rats

**DOI:** 10.1186/1476-511X-12-33

**Published:** 2013-03-13

**Authors:** Lin Yan, Xiao-long Bai, Zheng-feng Fang, Lian-qiang Che, Sheng-yu Xu, De Wu

**Affiliations:** 1 Key Laboratory for Animal Disease Resistance Nutrition of the Ministry of Education, and Animal Nutrition Institute of Sichuan Agricultural University, Ya’an, 625014, China

**Keywords:** Omega-3 fatty acids, Omega-6 fatty acids, Ratio, Male, Reproduction

## Abstract

**Background:**

Beneficial effects of omega-3 polyunsaturated fatty acids (n-3 PUFAs) on the reproduction of male animals are widely described in the literature. However, there is little information about the effect of n-3/n-6 PUFA ratios on male health and reproduction. The aim of this study was to investigate the effects of diets with different n-3/n-6 PUFA ratios on the reproductive performance of male rats.

**Methods:**

Eighty male Sprague Dawley (SD) rats were supplemented with diets containing different n-3/n-6 PUFA ratios (0.13, 0.40, 0.85, 1.52 and 2.85) for 60 days. Half of the rats in each group were sacrificed on day 60, and the other half were chosen to mate with female mice to assess the effects of n-3/n-6 ratios on reproductive performance.

**Results:**

Sperm density and sperm motility of the 1.52 group were higher than other groups (*P <* .05), and the development of testis and the morphological structure of sperm in the 1.52 group were better than other groups. Furthermore, a higher litter size and birth weights of offspring were observed in the 1.52 group. Additionally, serum reproductive hormone levels were significantly affected by the n-3/n-6 ratios.

**Conclusion:**

These findings demonstrated that a balanced n-3/n-6 ratio was important in male rat reproduction. Therefore there is a necessity to determine an appropriate n-3/n-6 PUFA ratio in man and different male animals in the future.

## Background

Sperm cells contain very high proportions of polyunsaturated fatty acids (PUFA) [1], and normal spermatozoa possess a higher percentage of the most representative PUFA (C22:6 n-3) than those detected in blood serum phospholipids and in other cell membranes [2]. The lipid composition, the degree of PUFA unsaturation, and the proportion of sperm PUFA have been shown to affect sperm quantity [2-4]. Animals cannot synthesize n-6 or n-3 fatty acids *de novo* because of a lack of the appropriate fatty acid desaturase enzymes. The n-6 PUFA and the n-3 PUFA therefore need to be provided in the diet as these PUFAs are essential for numerous processes including growth, reproduction, vision, and brain development [5].

Studies in men [4] and boars [1,6,7] have demonstrated the benefits of n-3 fatty acids on male reproductive capacity. While, other studies in humans [8] and boars [9] did not show any effect of n-3 PUFA supplementation on semen quality or quantity post-ejaculation. However, Am-in N [10] pointed out that the ratios of n-3/n-6 PUFAs in boar sperm were negatively correlated with sperm motility, viability, normal morphology, and normal plasma membranes, which suggested that the appropriate ratio of n-3/n-6 PUFAs in males was important for sperm quality. Although there is little information about the effect of n-3/n-6 ratios on male reproduction, other researchers have shown the beneficial role of an appropriate dietary n-3/n-6 ratio for embryo development and health. Dietary intake of a low ratio of n-6/n-3 PUFAs of about 1–2:1 during both maternal pregnancy and lactation may be more beneficial for early fetal development [11]. http://Santillán et al. [12] suggested that the maintenance of an adequate n-3/n-6 ratio was necessary for the optimal growth and development of murine offspring and several metabolic parameters in adulthood [13], including bone development [14]. Health benefits may be achieved by lowering dietary n-6/n-3 PUFAs even in a high fat diet medium [15]. However, modern dietary trends have increased this ratio from 10:1 to 25:1 in westernized human populations [16]. Therefore, in both human and animal diets there are grounds for maintaining the proper ratio of n-6 and n-3 PUFAs dietary intakes to promote reproduction. However, little is known about the effects of different ratios of n-6 and n-3 PUFAs on sperm quality and fertility, although both positive and negative actions are theoretically possible. Because of the lack of research data, additional research was needed. Thus, the objective of the present study was to determine the effects of different ratios of n-6 and n-3 PUFA intakes on semen quality, breeding ability, and serum reproductive hormone levels.

## Methods

### Animals and diets

All experimental protocols were approved by the Animal Care and Use Committee of the Sichuan Agricultural University, and were in accordance with the National Research Council’s Guide for the Care and Use of Laboratory Animals. Eighty male Sprague Dawley (SD) rats and forty female SD rats used in the present study were purchased from Chengdu Da Shuo Biotech Co., Ltd [License: SCXK 2008–24] with an initial age of 90-days old. Female rats were fed a commercial diet for mating with the males to further determine the effect of different ratios of n-3/n-6 PUFAs on litter size and birth weight of male rats’ offspring. Animals were housed individually in clear metallic cages within a bio-bubble in a temperature-controlled room (21–23°C) with a 12 h light/dark cycle.

The male rats were fed diets containing 7% oil from soybean and flaxseed for 60 days. The basic formulation of the experimental diets (Table 1) contained supplemental ratios of soybean oil (SO): flaxseed oil (FO), namely 100:0, 75:25, 50:50, 25:75, and 0:100. The ratios of dietary n-3/n-6 PUFAs were 0.13, 0.40, 0.85, 1.52, and 2.85. The fatty acid compositions of the oils used in this study are presented in Table 2 and the fatty acid compositions of the diets shown in Table 3. Rats were allowed free access to food and water.

**Table 1:** Composition and nutrient levels of basal diets

**Ingredients**	**Percentage(%)**
Corn starch	39.75
Casein	20.00
Gelatinization starch	13.20
Sucrose	10.00
Cellulose	5.00
Soybean oil	7.00
Linseed oil	0.00
Mineral premix ^1^	1.00
Vitamin premix ^2^	3.50
Choline Chloride	0.30
L-Cys	0.25
t-BHQ^3^	0.0014
Total	100.00

^1^ Provided per kg of diet: Calcium 5000 mg; Phosphorus 1561 mg; Kalium 3600 mg; Natrium 1019 mg; Chlorine 1517 mg; Magnesium 510 mg; Ferrum 35 mg; Znic 30 mg; Manganese 10 mg; Copper 6 mg; I 0.2 mg; Selenium 0.15 mg.

^2^ Provided per kg of diet: VD_3_ 1000 IU; VK_3_ 0.75 mg; VB_1_ 6.0 mg; VB_2_ 7.0 mg; VB_6_ 6.0 mg; VB_12_ 0.02 mg; nicotinic acid 30.0 mg; D-calcium pantothenate 15.3 mg; folic acid 2.0 mg; biotin 0.2 mg.

^3^ t-BHQ, tert-butyl hydroquinone.

**Table 2:** Fatty acid composition of soybean oil and flaxseed oil

**Fatty Acid**		**Soybean Oil**	**Flaxseed Oil**
Myristic(%)	14:0	0.06	0.03
Palmitic(%)	16:0	10.89	5.90
Palmitoleic(%)	16:1 n-7	0.14	0.11
Stearic(%)	18:0	3.25	4.36
Oleic(%)	18:1 n-9	24.5	22.96
Linoleic(%)	18:2 n-6	53.41	16.16
Linolenic(%)	18:3 n-3	7.34	49.93
Eicosanoic(%)	20:1 n-9	0.02	0.17
Eicosanoic(%)	20:1 n-7	0.04	0.17
Eicosapentaenoic(%)	20:5 n-3	—	—
Docosahexaenoic(%)	22:6 n-3	—	—
Other fatty acid(%)		0.35	0.21
Total fatty acid		100	100
Total n-3PUFAs		7.34	49.93
Total n-6PUFAs		53.41	16.16
n-3:n-6		1:7.30	1:0.32

Note: Values in the table are measured values (n = 4).

**Table 3:** Fatty acid composition of the diet(mg/g)

**Items**	**Ratio of SO:FO**				
SO:FO	100:0(Diet 1)	75:25(Diet 2)	50:50(Diet 3)	25:75(Diet 4)	0:100(Diet 5)
C12:0	0.07	0.06	0.07	0.07	0.04
C14:0	0.35	0.32	0.35	0.34	0.22
C14:1	0.01	0.01	0.01	0.01	0.01
C16:0	7.55	6.31	6.29	5.52	3.82
C16:1	0.11	0.10	0.10	0.10	0.08
C18:0	2.87	2.53	2.75	2.64	2.03
C18:1	0.27	0.23	0.24	0.23	0.16
C18:1n9	12.51	11.01	11.68	11.28	8.96
C18:1n7	0.80	0.65	0.62	0.53	0.37
C18:2n6	25.94	20.14	18.37	14.09	8.13
C18:3n6	0.29	0.01	0.02	0.32	0.29
C18:3n3	3.15	7.92	15.44	21.82	23.94
C20:0	0.22	0.18	0.17	0.14	0.08
C20:1	0.12	0.11	0.12	0.12	0.10
C20:2	0.05	0.04	0.04	0.03	0.02
C20:4n6	-	-	0.01	0.01	0.01
C20:3	-	0.01	0.02	0.02	0.02
C20:5n3	0.25	0.19	0.17	0.13	0.07
C22:0	0.04	0.02	0.03	0.02	0.02
C22:1	0.06	0.05	0.05	0.04	0.03
C22:4n6	0.01	0.01	0.01	0.02	0.02
C24:0	0.12	0.09	0.09	0.08	0.06
C22:5n3	0.04	0.02	0.03	0.02	0.02
C22:6n3	0.01	0.01	0.01	0.01	0.01
C24:1	0.03	0.02	0.01	0.01	0.01
Total n-3PUFAs	3.45	8.14	15.65	21.98	24.04
Total n-6PUFAs	26.24	20.17	18.41	14.44	8.44
Ratio of n-3:n-6	0.13	0.40	0.85	1.52	2.85

Note: Values in the table are measured values.

### Collection of blood and tissue samples

Rats were anesthetized in sealed jar containing cotton moistened by 350 μl isoflurane at 30 and 60 days. Tail vein blood was collected and centrifuged at 4000 rpm for 10 min at 4°C for the collection of serum and stored at −20°C until analysis.

### Semen quality and reproductive performance

Half of the rats in each treatment group were anesthetized and sacrificed for collection of testis, epididymis, and vas deferens on day 60. The epididymides were dissected in 2 ml normal saline (0.9% NaCl); incubated at 37°C, and sperm motility was evaluated by the procedures described in previous publication [17]. At least 200 spermatozoa and 5 fields were assessed for each specimen (n = 8), and the percentages of motile and progressively motile spermatozoa were determined. Sperm density was determined by using a hemocytometer count according to the method modified from that described by Taylor et al. [18] and Egbunike et al. [19]. Mean spermatozoa density was obtained by counting from 5 squares of a hemocytometer. Morphological abnormalities of the sperm head in 8 rats of every group were assessed essentially according to the criteria of Wyrobek and Bruce [20], and the results presented as percentages. Half of the male rats in each treatment group on day 61 were mated with female rats in a ratio of 1:1. All female rats were provided the same commercial standard chow during pregnancy. The pregnancy rate, litter size, and birth weight in the female rats were observed.

### Measurement of serum gonadotropin-releasing hormone (Gnrh), luteinizing hormone (LH), follicle stimulating hormone (FSH), and testosterone (T)

GnRH, LH, FSH and T concentrations in serum were measured using an ELISA kit (R&D Systems, Inc., Minneapolis, MN, USA) according to the manufacturer’s protocol. All assays were performed in 96-well plates and the absorbance was measured at 450 nm by using an enzyme-labeled meter (Thermo Electron Corporation, Varioskan TM, Waltham, MA, USA). The levels of hormones were determined from the standard curve and expressed as picograms per milligram protein.

### Lipid analysis

The fatty acid compositions of the diets (oils and treatment diets) were evaluated using HP6890 GC-FID gas chromatography by the Analysis and Testing Center of China Agricultural University according to a modified method [10].

### Testis and sperm electron microscopic examination

After male rats were anesthetized with ether, both testes of each rat were removed without epididymis and fresh weights taken. For electron microscopy, the right testis was fixed by perfusion with fixative containing 5% glutaraldehyde buffered with 0.16 mol/L s-collidine buffer, pH 7.4, for 15 min [21]. A 1 mm thick transverse slice vertical to the long axis of the testis was taken from the middle portion of the organ, cut into approximately 1 mm cubes and immersed in the same fixative for 2 h, washed with the same buffer, dehydrated in ethanol and embedded in Epon 812. A series of ultra-thin sections were cut on the LKB-Huxley ultramicrotome (LKB, Bromma, Sweden), with a diamond knife and stained with uranyl acetate and lead citrate,then examined and photographed using H-600 A-2 Jeol 1200 Ex-II electron microscopy (JEOL Ltd., Tokyo, Japan).

### Statistics

Data were analyzed using SPSS 11.5 statistical package (V8.1, SAS Institute Inc., Cary, NC) and presented as mean ± SEM. Multiple comparisons by DUNCAN analysis were performed to determine statistical differences among groups. Results were considered significant at *P* < 0.05 for all tests.

## Results

### Effects of different ratios of N-3/N-6 PUFAs on the weight gain and feed intake of male SD rats

As shown in Table 4, there were no significant differences in the initial body weights and average daily food intakes. Weight gain tended to increase as the ratio of n-3/n-6 PUFAs increased and the rats fed diets 4 and 5 had significantly (*P <* 0.05) higher weight gains when compared with rats fed diet 1 and diet 2.

**Table 4:** Effects of different ratio of N-3/N-6 on the weight gain and feed intake of male SD rats

**Items**	**Diet 1**	**Diet 2**	**Diet 3**	**Diet 4**	**Diet 5**
Ratio of n-3/n-6	0.13	0.40	0.85	1.52	2.85
Initial Body weight(g)	316 ± 12	316 ± 14	312 ± 12	315 ± 10	314 ± 13
Final weight(g)	388 ± 10^a^	395 ± 12^a^	405 ± 11^ab^	421 ± 15^b^	417 ± 12^b^
Weight gain(g)	71 ± 4^a^	79 ± 3 ^a^	92 ± 6^b^	106 ± 5^c^	103 ± 5^bc^
Food intake (g/day)	22.26 ± 2.04	22.13 ± 1.95	22.39 ± 2.11	23.32 ± 3.02	23.95 ± 2.48

Data were presented as means ± standard deviation (n = 16), and value in the same row with different superscript letters denotes significant difference (*p* < 0.05).

### Effects of different ratios of N-3/N-6 PUFAs on semen characteristics of male SD rats

There was no significant difference in testis index among groups (*P >* 0.05). Results of semen characteristics are shown in Table 5. The sperm density of the diet 4 group was significantly higher than the diet 1 and diet 3 groups (*P* < 0.05), but there was no significant differences between animals in the diet 2 and 5 groups. Meanwhile, the sperm motility in the diet 4 group was higher than other groups (*P* < 0.05), and there were no significant differences among diet groups 1, 2 and 3. All sperm head, mid-piece, and tail anomalies were considered sperm deformations. The sperm deformity ratio was significantly influenced by the ratio of n-3/n-6 PUFAs. The sperm deformity rate tended to decrease with the increasing n-3/n-6 ratio (*P <* 0.05), and was significantly lower in rats fed diets 4 and 5 compared with those fed other diets.

**Table 5:** Effects of different ratio of N-3/N-6 on semen characteristics of male SD rats

**Items**	**Diet 1**	**Diet 2**	**Diet 3**	**Diet 4**	**Diet 5**
Ratio of n-3/n-6	0.13	0.40	0.85	1.52	2.85
Testis index	11.22 ± 1.83	11.14 ± 1.17	10.74 ± 0.98	10.78 ± 0.80	10.54 ± 0.77
Sperm density (×10^6^ spz/mL)	41.00 ± 8.60^c^	50.00 ± 2.09^ab^	44.50 ± 5.28^bc^	54.67 ± 8.93^a^	50.83 ± 8.42^ab^
Motility (%)	66.67 ± 2.58^c^	66.33 ± 2.50^c^	68.83 ± 1.47^c^	82.33 ± 5.20^a^	74.50 ± 3.45^b^
Sperm deformity ratio (%)	8.47 ± 1.21^a^	8.65 ± 1.88^a^	6.68 ± 1.39^b^	3.16 ± 0.73^c^	4.17 ± 0.75^c^

Testis index = testis weight (mg)/ body weight (g).

Note: Data are presented as means ± standard deviation (n = 8), and means in the same row with different superscript letters denotes significant difference (*p* < 0.05).

### Effects of different ratios of N-3/N-6 PUFAs on histological and ultra-structural changes

**Figure 1:**
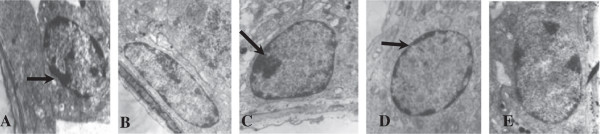
**Electron micrograph of a portion of testicular seminiferous tubules epithelium and spermatogonia of the epithelial bottom in rat testis.** ×80000; **A**, Diet 1 (0.13); **B**, Diet 2 (0.4); **C**, Diet 3 (0.85); **D**, Diet 4 (1.52); **E**: Diet 5 (2.85).

**Figure 2:**
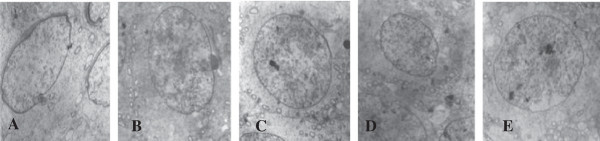
**Effects of different ratio of n-3/n-6 on spermatocytes histology of rats testis.** ×60000. **A**, Diet 1 (0.13); **B**, Diet 2 (0.4); **C**, Diet 3 (0.85); **D**, Diet 4 (1.52); **E**: Diet 5 (2.85).

As seen in Figure 1, testicular seminiferous tubules epithelium and spermatogonia of the underlying epithelial cells developed normally; there were chromatin clumps along the nuclear membrane. Compared with the control group, better spermatogonial development and more uniform distribution of chromatin around the nuclear membrane was observed in the group with a n-3/n-6 PUFA ratio of 1.52 (diet 4). Spermatocytes developed better than control group (Figure 2). The bridge between adjacent spermatocytes was visible and nucleoli were prominent, mitochondria abundant, and lysosomes observed in the cytoplasm in diets containing FO groups.

**Figure 3:**
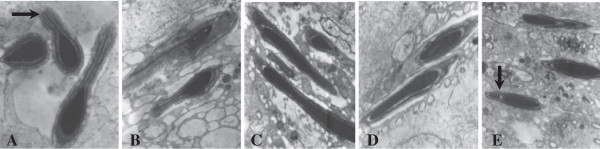
**Effects of different ratio of n-3/n-6 on sperm cells histology.** ×100000. **A**, Diet 1 (0.13); **B**, Diet 2 (0.4); **C**, Diet 3 (0.85); **D**, Diet 4 (1.52); **E**: Diet 5 (2.85).

**Figure 4:**
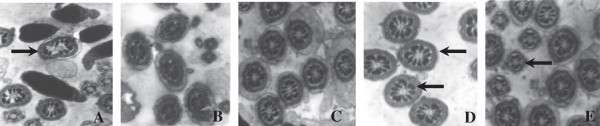
**Effects of different ratio of n-3/n-6 on cross sections of middle piece of sperm tails histology in rat testis.** ×150000. **A**, Diet 1 (0.13); **B**, Diet 2 (0.4); **C**, Diet 3 (0.85); **D**, Diet 4 (1.52); **E**: Diet 5 (2.85).

The structural integrity of mature sperm heads was presented in every group (Figure 3), while sperm tail abnormalities was more serious in the control group. Integral acrosomal cap and tail outer dense fibers and mitochondrial sheaths were observed in the testis in the group consuming a n-3/n-6 PUFA ratio of 1.52 (diet 4). Effects of the n-3/n-6 ratio on sperm tails histology are shown in Figure 4. The central axoneme, nine dense peripheral axonemal fibers, and the mitochondrial sheath in cross-sections of the middle piece of the sperm tails in the flaxseed oil supplemented groups were clearly visible compared with the control group, and were evenly distributed in the group consuming a n-3/n-6 PUFA ratio of 1.52.

### Effects of different ratios of N-3/N-6 PUFAs on reproductive capacity

Effects of n-3/n-6 PUFA ratios on reproductive capacity are shown in Table 6. The litter size in diet 1 group was the lowest, and improved with increasing n-3/n-6 levels (*P <* 0.05), while there was no significant difference between the ratio 1.52 and 2.85 groups, indicating the importance of an appropriate ratio of n-3/n-6 for normal reproductive function in male rats. A similar result was observed for birth weight, which was the lowest in diet 1 group and increased with the enhancing of the dietary n-3/n-6 PUFA ratio (*P <* 0.05).

**Table 6:** Effects of different ratio of N-3/N-6 on reproductive capacity of male SD rats

**Items**	**Diet 1**	**Diet 2**	**Diet 3**	**Diet 4**	**Diet 5**
Ratio of n-3/n-6	0.13	0.40	0.85	1.52	2.85
Litter size(n)	8.88 ± 1.75^c^	9.33 ± 1.03^bc^	9.17 ± 2.03^c^	12.3 ± 1.75^a^	11.33 ± 2.06^ab^
Birth weight (g)	5.83 ± 0.54^b^	5.93 ± 0.28^b^	6.22 ± 0.15^ab^	6.43 ± 0.39^a^	6.57 ± 0.28^a^

Note: Data are presented as means ± standard deviation (n = 8), and means in the same row with different superscript letters denotes significant difference (*p* < 0.05).

### Effects of different ratios of N-3/N-6 PUFAs on the serum hormone levels

The data for total circulating reproductive hormones are shown in Table 7. The GnRH concentrations were significantly higher in rats fed diet 4 compared with other groups on day 30, and increased with the increasing of the n-3/n-6 PUFA ratios on day 60 (*P <* 0.05). A similar trend was observed for LH, FSH, and T concentrations. The LH, FSH, and T concentrations in rats fed diet 4 were higher than other groups on days 30 and 60, and no difference was observed in groups fed diet 1, 2, and 3 (*P >* 0.05).

**Table 7:** Effects of different ratio of N-3/N-6 on the serum hormone levels of male SD rats

**Groups**	**GnRH(ng/L)**	**LH(ng/L)**	**FSH(IU/L)**	**T(nmol/L)**
30d				
Diet 1	10.14 ± 1.06^b^	17.37 ± 1.30^b^	5.76 ± 0.92^b^	40.23 ± 1.12^b^
Diet 2	10.07 ± 0.98^b^	17.76 ± 1.09^b^	5.55 ± 0.56^b^	40.29 ± 2.00^b^
Diet 3	10.45 ± 1.69^b^	18.43 ± 1.47^b^	6.50 ± 1.20^ab^	46.62 ± 8.06^b^
Diet 4	12.29 ± 0.46^a^	20.39 ± 0.55^a^	7.12 ± 0.99^a^	57.16 ± 4.06^a^
Diet 5	10.54 ± 1.56^b^	18.33 ± 1.57^b^	6.19 ± 0.86^ab^	45.81 ± 7.85^b^
60 d				
Diet 1	10.36 ± 1.30^c^	15.87 ± 0.96^c^	6.26 ± 0.48^b^	40.01 ± 1.73^c^
Diet 2	10.48 ± 0.74^bc^	16.04 ± 0.99^c^	6.17 ± 1.10^b^	42.36 ± 4.76^bc^
Diet 3	11.01 ± 1.60^abc^	16.08 ± 1.24^c^	5.57 ± 0.98^b^	39.86 ± 5.39^c^
Diet 4	11.93 ± 0.97^ab^	20.13 ± 0.93^a^	7.39 ± 1.06^a^	51.28 ± 3.82^a^
Diet 5	12.20 ± 1.51^a^	18.13 ± 1.76^b^	5.46 ± 0.46^b^	47.15 ± 5.25^ab^

GnRH, gonadotropin-releasing hormone; LH, luteinizing Hormone; FSH, follicle stimulating hormone; T, testosterone.

Note: Data are presented as means ± standard deviation (n = 6), and means in the same columns with different superscript letters denotes significant difference (*p* < 0.05).

## Discussion

The purpose of this study was to evaluate the impact of different ratios of n-3/n-6 PUFAs on sperm quality and reproductive performance, and provide the basis for determining the appropriate n-3/n-6 PUFA ratio for male animals. Accumulating evidence from studies in men [4,22] and animals [1,6,7] indicated that consumption of n-3 fatty acids was beneficial to male reproductive capacity, but the appropriate ratio of n-3/n-6 PUFAs for male reproduction was still not known.

The results of this study showed that different ratios of n-3/n-6 PUFAs had no effects on the testis index, but improved sperm quality. With an increasing n-3/n-6 PUFA ratio, sperm density and motility were increased, and the sperm deformity rate tended to decrease. Additionally, better histological and ultra-structural changes of testis and sperm were observed in the group consuming a n-3/n-6 PUFA ratio of 1.52. It was reported that boar diets supplemented with 30 g/kg of tuna oil or fish oil (rich in long chain n-3 fatty acids) increased sperm motility and the content of normal acrosome and sperm cell morphology [1], improving the total number of sperm per ejaculation, and the membrane integrity of sperm [6]. More recently, it was found that boar diets fortified with n-3 rich fatty acid additives enhanced the sperm total number of average ejaculations, and the morphological integrity of sperm was improved [7]. Furthermore, the ratio of n-3/n-6 PUFAs in boar sperm were positively correlated with sperm motility, viability, normal morphology, and normal plasma membranes [10], and excessive n-3 PUFA supplementation decreased the sperm density and motility in our experiment, which indicated the importance of the n-6/n-3 PUFA ratio in sperm quality.

Research has shown that diets containing distinct lipid sources differentially modified the lipid contents of the sperm head and body membranes, resulting in significant improvement in semen quality [23,24]. Al-Daraji et al. [23] found the proportion of n-3 fatty acids in spermatozoa from Japanese male quail fed fish oil compared with corn oil was higher (9.6% vs. 4.3%) and that of n-6 fatty acids was lower (22.4% vs. 33.3%). The sperm of flaxseed-fed rabbits had an n-3/n-6 ratio two times higher compared with the control because of the increasing dietary n-3/n-6 ratio [25]. In addition, it was reported that diets containing different lipid sources changed the lipid contents of sperm, mainly affecting the sperm head and body membranes [24]. It is worth noting that the dietary n-3/n-6 PUFA ratio affected the lipid composition of perch semen. However, no significant effects of changes in the n-3/n-6 ratios were observed in the sperm volume density and spermatozoa motility when the n-3/n-6 ratios were 0.2 and 7.0 respectively [26], indicating high correlations between these changes in dietary lipid content and sperm lipid concentration, and that an appropriate n-3/n-6 PUFA ratio was important for sperm quality. Therefore, we speculate that a possible reason for improving sperm quality in our experiment should be related to the changes in sperm composition induced by different fatty acid compositions in the diets.

In fact, very few studies have been conducted to examine the effects of the ratio of n-3/n-6 PUFAs on male reproduction. It is known that both n-6 and n-3 PUFAs can influence reproductive processes. In this study, we found that litter size and birth weight increased with increasing n-3/n-6 PUFA ratios; lower or higher n-3/n-6 ratios have adverse effects on reproduction, which was consistent with the results of sperm quality and sperm morphology. Blesbois [27] found that increasing the ratio of n-3/n-6 PUFAs in the diet can enhance the hatching rates of male turkeys at 48–58 weeks by nearly 2 points. Higher rates of embryonic and larval survival were observed in male European sea bass fed PUFA-enriched diets [28]. Similarly, studies conducted in females also have observed positive effects of diets rich in n-3 PUFA on reproductive performance. It was demonstrated that supplementing n-3 fatty acids from fish oil in the diet of sows improved early embryo survival [29], thereby increased piglet litter sizes [30,31]. Meanwhile, the content and variety of the maternal intake of PUFAs were shown to be associated with weight gain and growth of infants, particularly in preterm infants [32,33]. Therefore, these results confirmed that maintaining an appropriate n-3/n-6 PUFA ratio was very important for reproductive performance in males and females.

Twenty-carbon PUFAs are the direct precursors of a large group of physiologically active compounds [34]. From our results, we found that by increasing the ratio of n-3/n-6 PUFAs, the concentrations of GnRH, FSH, LH, and T increased. However, levels of these hormones dropped when the n-3/n-6 PUFA ratio was over 1.52, suggesting a close relationship between the n-3/n-6 PUFA ratio and the reproductive endocrine system. In males, the hypothalamus secretes GnRH, which binds to GnRH receptors on the gonadotropic cells to stimulate the release of FSH and LH into the circulation. LH stimulates the interstitial cells located in the testes to produce testosterone, and FSH plays a role in spermatogenesis [35]. The reduction of LH pulse frequency of mature male sheep after reduction of feed intake has been observed [36], while putting rams on a high energy diet increased GnRH pulse frequency, testicular mass, and sperm production [37]. The increase of LH and FSH levels in rats after supplementation with *Nigella sativa* oil may be the result of a direct effect of the oil on the hypothalamus, which in turn increases GnRH [38]. Similar effects also were observed on levels of plasma testosterone, which were higher when the same diet was available *ad libitum* than in restricted growth lambs from 7 to 17 weeks. *Ad libitum* feeding stimulated increased plasma FSH and LH levels above values for the restricted growth group [39]. Bubenik et al. [40] reported that LH levels in high-protein/low-energy fed deer were higher than those fed high-protein/high-energy diets in summer. Furthermore, lower energy intakes during pregnancy significantly reduced peak levels of testosterone in deer male offspring [40], suggesting that nutrition not only affected the reproductive hormone secretion and reproduction function of the male animal, but also may affected reproductive development and function in future generations.

Modern dietary trends have increased the n-6/n-3 PUFA ratio from 10:1 to 25:1 in westernized human populations [16]. http://Tian et al. [11] suggested that a diet with a low ratio of n-3/n-6 PUFAs at about 0.5-1:1 during both maternal pregnancy and lactation may be more beneficial for early development; our results regarding the ratio of n-3/n-6 PUFA at 1.52 are consistent with the previous findings. Other studies have reported that diets with high n-6/n-3 PUFA ratios may enhance the risk for both depression and inflammatory diseases [41], while a low ratio of n-6/n-3 PUFAs improved growth and development of murine offspring [13], as well as several metabolic parameters in adulthood [14] and human health [16,19]. Results of this study clearly indicate that the ratios of n-3/n-6 PUFAs in the diet have a great influence on sperm quality traits and reproductive performance, and that a n-3/n-6 PUFAs ratio of 1.52 improved the reproductive capacity of male rats.

## Conclusions

In conclusion, intake of an appropriate n-3/n-6 PUFA ratio in the diet of rats increased sperm characteristics and enhanced the structure integrity of testis and sperm, thereby improving reproductive performance, which may be related to changes in hormone metabolism. These findings provide a sound basis that a balanced n-3/n-6 PUFA ratio will be beneficial to male reproduction. Therefore there is a necessity to determine an appropriate n-3/n-6 PUFA ratio in man and different male animals in the future.

## Abbreviations

n-3/n-6 PUFA: Omega-3/omega-6 polyunsaturated fatty acid; GnRH: Gonadotropin-releasing hormone; LH: Luteinizing hormone; FSH: Follicle stimulating hormone; T: Testosterone.

## Competing interests

The authors declare that they have no competing interests.

## Authors’ contributions

YL was involved in the study, experimental design, data analysis, and manuscript writing. XLB was involved in the experimental design and animal experiments. ZFF was involved in the experimental design and data analysis. LQC was involved in study design and data interpretation. SYX was involved in data analysis. The group leader of the Institute of Animal Nutrition, DW, was involved in the study design and manuscript editing. All authors have read and approved the final manuscript.
